# Predictive Modeling of Signaling Crosstalk during C. elegans Vulval Development

**DOI:** 10.1371/journal.pcbi.0030092

**Published:** 2007-05-18

**Authors:** Jasmin Fisher, Nir Piterman, Alex Hajnal, Thomas A Henzinger

**Affiliations:** 1 School of Computer and Communication Sciences, Swiss Federal Institute of Technology (EPFL), Lausanne, Switzerland; 2 Institute of Zoology, University of Zurich, Zurich, Switzerland; 3 Electrical Engineering and Computer Science, University of California Berkeley, Berkeley, California, United States of America; The University of Tokyo, Japan

## Abstract

Caenorhabditis elegans vulval development provides an important paradigm for studying the process of cell fate determination and pattern formation during animal development. Although many genes controlling vulval cell fate specification have been identified, how they orchestrate themselves to generate a robust and invariant pattern of cell fates is not yet completely understood. Here, we have developed a dynamic computational model incorporating the current mechanistic understanding of gene interactions during this patterning process. A key feature of our model is the inclusion of multiple modes of crosstalk between the epidermal growth factor receptor (EGFR) and LIN-12/Notch signaling pathways, which together determine the fates of the six vulval precursor cells (VPCs). Computational analysis, using the *model-checking* technique, provides new biological insights into the regulatory network governing VPC fate specification and predicts novel negative feedback loops. In addition, our analysis shows that most mutations affecting vulval development lead to stable fate patterns in spite of variations in synchronicity between VPCs. Computational searches for the basis of this robustness show that a sequential activation of the EGFR-mediated inductive signaling and LIN-12 / Notch-mediated lateral signaling pathways is key to achieve a stable cell fate pattern. We demonstrate experimentally a time-delay between the activation of the inductive and lateral signaling pathways in wild-type animals and the loss of sequential signaling in mutants showing unstable fate patterns; thus, validating two key predictions provided by our modeling work. The insights gained by our modeling study further substantiate the usefulness of executing and analyzing mechanistic models to investigate complex biological behaviors.

## Author Summary

Systems biology aims to gain a system-level understanding of living systems. To achieve such an understanding, we need to establish the methodologies and techniques to understand biological systems in their full complexity. One such attempt is to use methods designed for the construction and analysis of complex computerized systems to model biological systems. Describing mechanistic models in biology in a dynamic and executable language offers great advantages for representing time and parallelism, which are important features of biological behavior. In addition, automatic analysis methods can be used to ensure the consistency of computational models with biological data on which they are based. We have developed a dynamic computational model describing the current mechanistic understanding of cell fate determination during C. elegans vulval development, which provides an important paradigm for studying animal development. Our model is realistic, reproduces up-to-date experimental observations, allows in silico experimentation, and is analyzable by automatic tools. Analysis of our model provides new insights into the temporal aspects of the cell fate patterning process and predicts new modes of interaction between the signaling pathways involved. These biological insights, which were also validated experimentally, further substantiate the usefulness of dynamic computational models to investigate complex biological behaviors.

## Introduction

Describing mechanistic models in biology in a formal language, especially one that is dynamic and executable by computer, has recently been shown to have various advantages (see review [1]). A formal language comes with a rigorous semantics that goes beyond the simple positive and negative interaction symbols typically used in biological diagrammatic models. If the language used to formalize the model is intended for describing dynamic processes, the semantics, by its very nature, provides the means for tracing the dynamics of system behavior, which is the ability to run, or execute, the models described therein.

Dynamic models can represent phenomena of importance to biological behaviors that static diagrammatic models cannot represent, such as time and concurrency. In addition, formal verification methods can be used to ensure the consistency of such computational models with the biological data on which they are based [2,3]. It was previously suggested that by formalizing both the experimental observations obtained from a biological system and the mechanisms underlying the system's behaviors, one can formally verify that the mechanistic model reproduces the system's known behavior [3].

Formal models are used in a variety of situations to predict the behavior of real systems and have the advantage that they can be executed by computers; often at a fraction of the cost, time, or resource consumption that the observation of the real system would require. In addition, formal models have the advantage that they can be analyzed by computers. For example, it may be possible to predict, by analyzing a model, that all possible executions will reach a stable state, independent of environment behavior. The result of such an analysis would not be obtainable by executing the real system, no matter for how long or how many times, as there are often infinitely many possible environment behaviors. This process of computational model analysis, in the case of state-based models, is called *model checking* [4].

Here we follow the idea that model execution and model checking can be used to test a biological hypothesis: if the execution and analysis of the model conform to the experimental observations of the biological system, then the model may correctly represent the mechanism that underlies the system behavior; otherwise, the model needs modification or refinement. Thus, the model can be seen as a “hypothesis,” i.e., an explanation for a biological mechanism and experiments can confirm or falsify the hypothesis.

As part of an ongoing effort to model C. elegans vulval development [3,5], we have previously created a formal dynamic model of vulval cell fate specification based solely on the proposed diagrammatic model of Sternberg and Horvitz from 1989 [6]. Our previous work [3] has demonstrated that state-based mechanistic models are particularly well-suited for capturing the level of understanding obtained using the tools and approaches common in the field of developmental genetics, and that creating such executable biological models is indeed beneficial. Since the original model proposed by Sternberg and Horvitz (1989), our understanding of the molecular pathways governing vulval fate specification has advanced significantly. In particular, several modes of crosstalk and lateral inhibition between the major signaling pathways specifying the vulval cell fates have been discovered. Here, we report on a dynamic computational model of the more sophisticated understanding of vulval cell fate specification that we have today. In addition, we use model checking to test the consistency of the current conceptual model for vulval precursor cells (VPC) fate specification with a large set of observed behaviors and experimental perturbations of the vulval system.

The C. elegans vulva is formed by the descendants of three VPCs that are members of a group of six equivalent VPCs named P3.p–P8.p (Figure 1). Each of the six VPCs is capable of adopting one of three cell fates (termed 1°, 2°, or 3°) [6–8]. The actual fate a VPC adopts depends upon the integration of two opposing signals that each VPC receives: an inductive signal emanating from the gonadal anchor cell (AC) in the form of the LIN-3 epidermal growth factor activates the epidermal growth factor receptor (EGFR)/LET-23 in the VPCs. The inductive signal is transduced downstream of the EGFR/LET-23 by the conserved RAS/MAPK signaling cascade to specify the 1° cell fate. In response to the inductive signal, the VPCs produce a lateral signal that counteracts the inductive AC signal in the neighboring VPCs (lateral inhibition) by inducing the expression of a set of inhibitors of the EGFR/RAS/MAPK pathway collectively termed *lst* genes (for lateral signal targets) [9–11]. In a second step, the lateral signal induces the 2° cell fate (lateral specification) [12]. The lateral signal is encoded by three functionally redundant members of the Delta/Serrate protein family (*dsl-1, apx-1,* and *lag-2*) and transduced by the LIN-12 / Notch receptor [13,14]. Moreover, two functionally redundant inhibitory pathways defined by the synthetic Multivulva (synMuv) genes prevent the surrounding hypodermal syncytium (hyp7) from producing the inductive LIN-3 signal, thus allowing the AC to establish a gradient of inductive LIN-3 signal [15]. The fate of the VPCs is influenced by their relative distance from the AC and the lateral signals between the VPCs. The cell closest to the AC (P6.p) receives most of the inductive signal and adopts the 1° fate. The neighboring cells P5.p and P7.p receive the stronger lateral signal from P6.p and hence adopt the 2° fate. The remaining distal VPCs P3.p, P4.p, and P8.p adopt the 3° fate as they do not receive enough inductive signal or lateral signal; and LIN-3 expression in the hyp7 is repressed by the synMuv genes [8,15–17].

**Figure 1:**
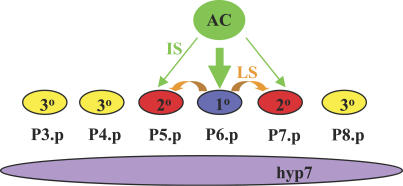
Signaling Events Involved in VPC Fate Specification IS, inductive signal; LS, lateral signal.

One remarkable feature of vulval fate specification is its absolute precision. Despite the ability of each cell to adopt any of the three cell fates, the pattern of fates adopted by P3.p–P8.p in wild-type animals is always 3°-3°-2°-1°-2°-3°, respectively. This precision is thought to be achieved by multiple modes of crosstalk between the inductive and lateral signaling pathways discovered in recent years [6,9–11,18,19].

Here we use computer simulations and formal verification to investigate whether the known gene interactions are sufficient to produce such patterning precision and to gain insights into the system dynamics. Using the language of *Reactive Modules* (RM) [20] and the *Mocha* tool [21], we have constructed a discrete, dynamic, state-based mechanistic model consisting of the key components of the inductive and lateral signaling pathways with their interconnections. By looking analytically at all possible behaviors of a model, we find previously unnoticed dependencies that are present in the data and explained by the model. Specifically, the analysis of our model predicts additional genetic interactions necessary for efficient lateral inhibition and, through the analysis of the behavior of *lin-15* mutants, gives new insights into the temporal order of events necessary to achieve a stable pattern of cell fates, which were also validated experimentally.

## Results

### Model Construction

Models in the language of RM are constructed by defining the objects of the system (these are the modules), and their variables representing semi-independent components of an object. The state of the system is determined by the states of its objects, which in turn are determined by the values of all their variables. Changes in the value of a variable depend on the previous values of the variable and possibly on other variables. A behavior of the system is a sequence of states that the system goes through during execution.

Our model consists of a worm module that comprises an AC module and six identical copies of a VPC module (Figure 2). Additional modules handle the synchronization between VPCs (i.e., the scheduler in Figure 2, which is setting the order of interaction between the VPCs for a particular execution) and manage the initialization of simulations (i.e., the organizer in Figure 2, which is setting the initial conditions for a particular execution). Each VPC module runs its own copy of the same program simultaneously, based on the inputs it receives from its neighboring cells (AC, hyp7, and the adjacent VPCs) (Figure 3). All VPCs begin with the same conditions determined by the genetic background but may receive different levels of inductive signal depending on their distance from the AC.

**Figure 2:**
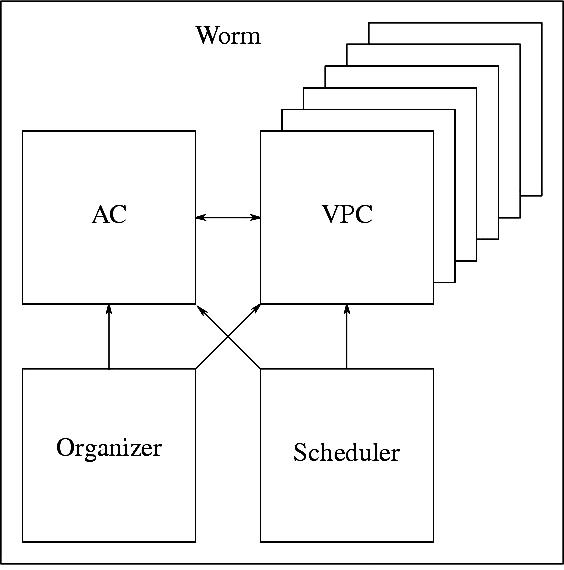
Modules Composing the Worm Vulva Model Communication between modules is marked by arrows. Communication between VPCs is not depicted.

**Figure 3:**
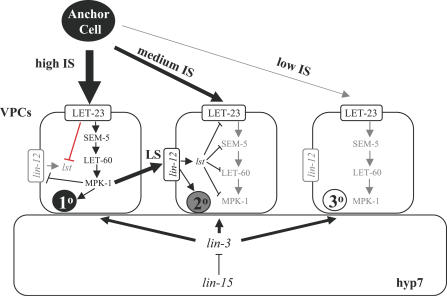
Conceptual Model for the Signaling Events Underlying VPC Fate Specification The thickness of the inductive signal (IS) arrows indicates the relative levels received by the three VPCs shown. With a low level of IS (the rightmost cell), the EGFR/RAS/MAPK pathway is below the threshold needed for induction (indicated in grey), and the VPC adopts the 3° fate. A high level of IS (the leftmost cell) activates the EGFR/RAS/MAPK pathway and induces the 1° fate. High IS also results in the production of a strong lateral signal (LS) by this VPC, the downregulation of LIN-12, and, as predicted by our computational model (see below), in the inhibition of the *lst* genes as indicated by the red line. The middle VPC receives a medium IS; however, the EGFR/RAS/MAPK pathway is counteracted (indicated in grey) by the *lst* genes activated by the strong LS from the leftmost VPC. The middle VPC thus adopts the 2° fate.

The AC module contains variables that indicate if the AC is ablated or formed and determine the level of inductive signal sensed by the VPCs according to their distance from the AC. If the AC is ablated, the inductive signal variables in all VPCs are set to the OFF level. If the AC is not ablated, the VPC closest to the AC (P6.p) senses HIGH inductive signal, the next closest (P5.p and P7.p) sense MEDIUM inductive signal, and the farthest (P3.p, P4.p, and P8.p) sense LOW inductive signal (Figure 3).

The VPC module contains variables that represent the behavior of the EGFR/RAS/MAPK pathway, the LIN-12 Notch-mediated lateral signaling pathway, and the *lin*-15–mediated inhibition of LIN-3 EGF in hyp7 (Figure 4). In addition, there is a variable for each VPC that follows the temporal progress toward fate acquisition. Each of these variables is now described briefly:

**Figure 4:**
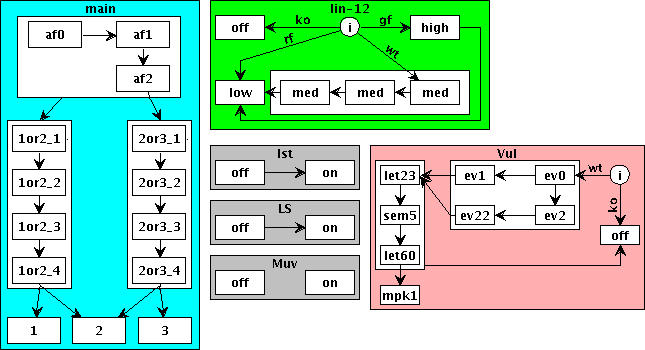
Graphic Visualization of the VPC Module Each rectangle represents a possible value, and arrows represent possible value changes (according to conditions on values of other components). The “main” component follows the progress of the cell toward fate assumption. The cell starts as undifferentiated (af); according to the activities in the EGFR and LIN-12 / Notch pathways, it decides whether to adopt a vulval (1or2) or a nonvulval (2or3) cell fate. Finally, the cell assumes one of the three cell fates. The smaller rectangles correspond to time delays until these decisions are made. Other components represent the activity level of the biological components they are associated with (*lin*-12, EGFR pathway, *lst* genes, LS, *lin*-15). Our tool also enables visualization of executions of this model by highlighting the current value of each component. Changes in highlighted values allow us to follow the execution visually.

The lateral signal variable (LS) can be either ON or OFF. The variable starts as OFF and is turned ON upon activation by the EGFR/RAS/MAPK pathway. Once the lateral signal is ON, it is sensed by the immediate neighbors of the respective VPC.

The *lin-*12 variable represents the level of *lin-*12 */ Notch* activity. If *lin-*12 activity is specified as wild-type, its activity level starts as MEDIUM in all VPCs. If *lin-*12 activity is eliminated [*lin-*12(0) mutation], then the *lin-*12 variable is set to OFF (when using the word *set* we mean that its value cannot change). By contrast, increasing *lin-*12 activity [*lin-*12(*d*) mutation] leads the variable to start as HIGH. Upon activation by the lateral signal, *lin-*12 activity increases from MEDIUM to HIGH. Upon inhibition of *lin-*12 activity by the EGFR/RAS/MAPK pathway, *lin-*12 activity decreases from MEDIUM or HIGH to LOW.

The *lst* genes variable, which can be either ON or OFF, collectively represents the activation state of the *lst* genes *lip-*1, *ark-*1, *dpy-*23, and *lst*-1 through *lst-*4 [9–11]. If *lst* genes are mutated to an inactive state, the variable is set to OFF. If all *lst* genes are wild-type, the variable starts as OFF and switches to ON upon activation by *lin-*12. We consider all the *lst* genes either as wild-type or as mutated to an inactive state.

The *lin-*15 variable collectively represents the state of *lin-*15 and other synMuv genes in hyp7, which can be either ON or OFF. If *lin-*15 is wild-type, this variable is set to ON and constitutively inhibits EGFR activation by LIN-3 from hyp7 (Figure 3). On the other hand, if *lin-*15 is mutated to an inactive state, the *lin-*15 variable is set to OFF, and the EGFR/RAS/MAPK pathway is constitutively and uniformly activated in all VPCs.

The inductive EGFR/RAS/MAPK pathway is represented by variables describing the status of the following four core components: *let*-23 (the EGF receptor), *sem*-5 (the Grb2-like adaptor), *let*-60 (the RAS GTP-binding protein), and *mpk*-1 (the MAP kinase). We consider either the wild-type behavior or mutations that completely inactivate a component (e.g., *let*-23(0) mutation causing the rest of the pathway never to be activated). Before the inductive signal is produced, *let*-23 *egfr* is OFF in all VPCs due to the presence of *lin*-15 and other synMuv genes, which prevent ectopic activation of LET-23 by repressing *lin-*3 *egf* expression in hyp7 [15]. Upon receiving the inductive signal from the AC, the variables simulate the activation of *let*-23, then *sem*-5, then *let*-60, and then *mpk-*1. The activation by a MEDIUM inductive signal is slower than the activation by a HIGH inductive signal. The EGFR/RAS/MAPK pathway can be counteracted by the *lst* variable described above during every stage of this activation sequence (see Figure 3, middle VPC).

### Model Validation

A simulation starts by setting the type of mutation(s) that we would like to examine, and then following an execution of the model by choosing how to schedule the different VPCs. Once the cells assume their fates, we compare the fate assumption versus the desired experimental results.

To capture the diverse behavior often observed in biological systems, such as cases where the same genotype leads to different fate patterns, we allow complete freedom in the order of reactions between the different VPCs modules, but restrict the amount of progress each cell makes before its neighbors. The resulting model is highly nondeterministic, allowing many choices of execution without giving priorities or quantities to each choice. Each VPC is treated as a separate process. By adding a mechanism that decides which VPC to advance and for how long, we could get various patterns of VPC fates in different executions. Consequently, the model has approximately 4,000 different possible ways to complete one round in which all cells move. Subsequently, there are about 10^36^ possible executions of the model. In addition, the model has 48 initial states, corresponding to 48 different experimental conditions, and about 92,000 different reachable states (possible assignments to all the variables), each corresponding to a snapshot of the system.

As the number of possible executions of the model is astronomical, we use formal verification to ensure that *all* possible runs of the model emanating from a given mutation produce results that match the experimental results. To do that, we have formalized the experimental observations that led to formulate the mechanistic model underlying VPC pattern formation (e.g., if the model starts in the wild-type state, the VPCs assume fates according to the following pattern: 3°-3°-2°-1°-2°-3°), and used them to formally check whether the mechanistic model reproduces the reported experimental observations. Once we have established a model that reproduces all the experimental data, we can also use simulations to predict the outcome of new experiments that have not been performed yet.

### Model Analysis

In nondeterministic models, simple simulations (i.e., testing) are not sufficient to verify the model's consistency with the experimental data. The reason is that in nondeterministic models the number of possible behaviors resulting form the same initial condition could be enormous. Therefore, to test a nondeterministic model, one would have to run many simulations (one for each scenario). Another way to test nondeterministic models is to use *model checking* [4], which allows us to formally check *all* the different executions of the system against a formal specification. By exploring all the possible states and transitions of a system, we can determine whether some property holds true for the system. In the case that the property does not hold, the model-checking algorithm supplies a “counterexample,” which is an execution of the system that does not satisfy the given requirement, in the current case an experimentally observed pattern of vulval cell fates.

Here, we have used model checking for two purposes. First, to ascertain that our mechanistic model reproduces the biological behavior observed in different mutant backgrounds. For that, we have formalized the experimental results described in a set of papers (for references see Table 1) and verified that all possible executions satisfy these behaviors. That is, regardless of the order of interactions from a given set of initial conditions, different executions always reproduced the experimental observations. Second, we used model checking to query the behavior of the model. By phrasing queries such as which mutations may lead to a stable or an unstable fate pattern, we analyze the behavior of the model. Once an unstable mutation was found, we determined what part of the execution allows this kind of mutation by disallowing different behavioral features of the model and checking when the instability disappears.

**Table 1:**
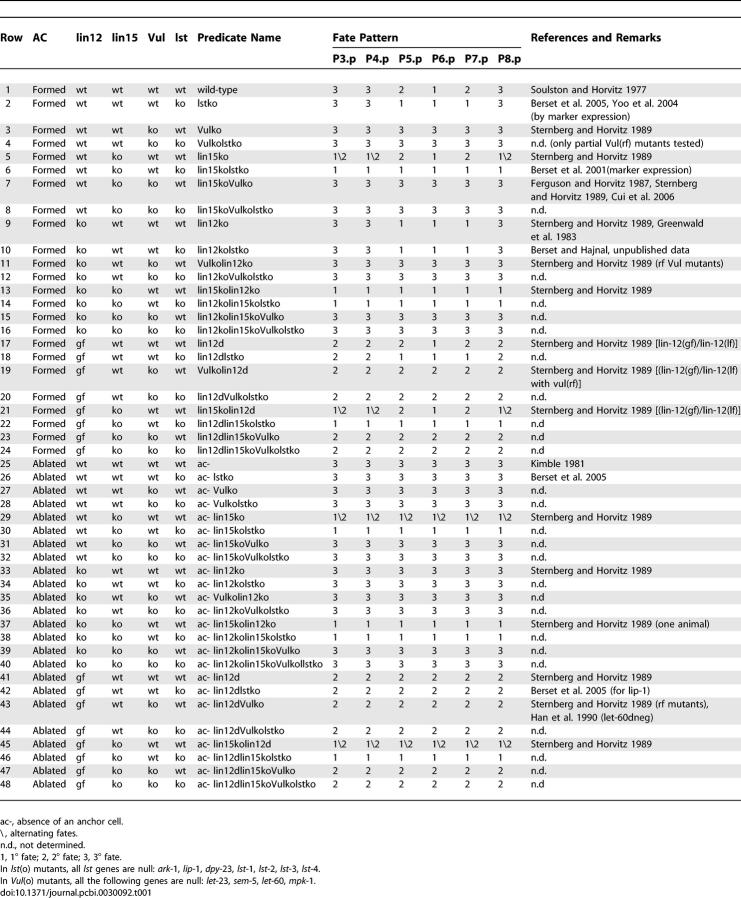
Summary of VPC Fate Patterns According to the Computational Model

### Gaining Insights into the Mechanism of VPC Fate Specification: A New Putative Negative Feedback Loop

We have tested the behavior of our model for a set of 48 perturbations corresponding to 24 mutant combinations, which were analyzed in the presence and absence of the AC (Table 1). For some of the combinations, the outcome has been tested experimentally as indicated in Table 1 by the respective references, but many other combinations have not been tested experimentally, as some of the double, triple, or quadruple mutants might be technically very difficult to generate. For example, complete loss-of-function mutations in most components of the inductive signaling pathway cause early larval lethality, or homozygous *lin-*12(*gf*) mutants lack an AC.

Forty-four of the 48 conditions tested yielded a stable fate pattern, as all possible executions gave the same result. All four conditions leading to an unstable pattern included the *lin*-15 knockout mutation. The cause of the unstable pattern in these four cases is discussed in the next section. Twenty-two of the conditions have been tested experimentally and the observed results are reported in the literature (see references in Table 1). Our model faithfully reproduces the predominant cell fate patterns that had been reported except for the phenotype of *lin*-12(*d*); *lin-*15(0) double mutants. While in *lin-*12(*d*); *lin*-15(0) animals, the distal VPCs (P3.p, P4.p, and P8.p) adopt either a 1° or a 2° cell fate [6], our model predicted that the six VPCs would always adopt a 2° fate. This discrepancy was traced to the fact that the high activity of LIN-12 simultaneously induced *lst* expression in all VPCs, which immediately repressed the transduction of the EGFR signal that was activated in all VPCs due to the *lin*-15(0) mutation (Figure 5A). The high levels of *lst* gene expression thus prevented the cells from engaging the mechanisms reducing LIN-12 activity, which is necessary for a 1° fate specification. In spite of having represented LIN-12 downregulation by EGFR signaling [18] and *lst*-mediated lateral inhibition on EGFR signaling [9,10], the model could not reproduce the experimental observations. Therefore, we postulated that some additional regulation is needed to allow primary fates while avoiding adjacent primary fates [22]. One possibility is that EGFR signaling downregulates one or several *lst* genes in addition to inducing *lin*-12 degradation (Figure 5B). If this happens before the activation of the *lst* genes completely blocks EGFR/RAS/MAPK signaling, then at least some VPCs are allowed to adopt a 1° fate. We further suggest that in order to avoid adjacent primary cells in these *lin-*12(*d*); *lin*-15(0) mutants, the lateral signal can still override the EGFR signal and *lst* activity prevails.

**Figure 5:**
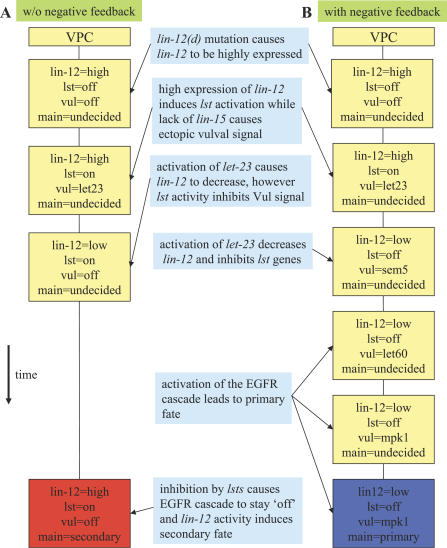
Simulation of the Model in the Absence (A) and Presence (B) of a Negative Feedback from EGFR to *lst* Genes Each rectangle represents a snapshot of the state of a VPC. Time flows from top to bottom and the changes in values of components represent the evolvement of simulation. Both simulations are according to the mutation *lin*-12(*gf*);*lin*-15(o). Both simulations start with *lin*-12 activated (according to *lin*-12(*gf*) mutation), and *let*-23 activated (according to *lin*-15(o), no inhibition from hyp-7). (A) *lin*-12 activates *lst*. Activation of *let*-23 inhibits *lin*-12; however, activation of *lst* prevents activation of *sem*-5. EGFR is counteracted and the cell assumes a 2° fate (red). (B) Inhibition of *let*-23 on *lst* prevents *lst* activation. The EGFR pathway is fully activated and the cell assumes a 1° fate (blue).

These insights led to a revised model with at least one additional negative feedback loop indicated by the red line in Figure 3. This refined model reproduces all the experimentally observed cell fate patterns including the *lin-*12(*d*); *lin*-15(0) double mutants (Table 1, rows 21 and 45). Of particular interest are those cases where two signaling pathways specifying different cell fates are simultaneously perturbed. For example, if the lateral signal is constitutively activated and at the same time the transduction of the inductive signal is blocked, then all VPCs are predicted to adopt the 2° cell fate irrespective of the presence or absence of the AC (Table 1, rows 19 and 43). Indeed, in *lin-*12(*n*137*gf*) mutants that carry a dominant-negative or strong reduction-of-function mutation in *let-*60*/Ras,* all VPCs were found to adopt the 2° cell fate [23]. In another condition we examined the interaction between the inhibitory *lin*-15 pathway and the *lst* genes. If both components are inactivated at the same time, all the VPCs are predicted to adopt a 1° cell fate in the presence as well as in the absence of the AC (Table 1, rows 6 and 30). As predicted by modeling, in the majority of *lin*-15(*n*309); *lip*-1(*zh*15) double mutants, adjacent VPCs adopt the 1° cell fate indicated by the expression of the 1° fate marker *egl-*17::*gfp* and by morphological criteria ([9] and unpublished data). An example for a condition that could not be tested experimentally is shown in Table 1, row 38. If all three signals, the inductive and lateral signals as well as the *lin*-15–mediated inhibition of hyp7, are inactive and the *lst* genes are mutated, all six VPCs are predicted to adopt the 1° cell fate as long as the EGFR/RAS/MAPK pathway is functional. This suggests that the default fate in the vulval equivalence group is 1°. Conversely, if the inductive and lateral signaling pathways are both constitutively activated (“lin15kolin12d” in Table 1, row 21), then the VPCs may adopt a 1° or 2° fate depending on the activity state of the *lst* genes (Table 1, rows 21, 22, 45, and 46).

In summary, by using model checking to compare our executable model with existing experimental data, we can predict novel interactions in the regulatory network governing vulval fate specification. In addition, analysis of the model allows us to predict the outcome of perturbations that are difficult to test experimentally.

### Sequential Signaling Is Necessary for Stable Pattern Formation

Using model checking, we found that 44 out of 48 perturbations affecting vulval development lead to a stable fate pattern, despite the vast number of possible executions of our model. The only four mutations leading to unstable patterns are *lin-*15(0), *lin-*15(0); *lin*-12(*d*), *lin*-15(0); *ac*- and *lin*-15(0); *lin*-12(*d*) and *ac*- (Table 1, rows 15, 21, 29, and 45). To determine whether variations in the exact timing of the lateral signaling are the cause of this instability, we asked, using model checking, whether it is possible to get an unstable fate pattern without allowing variations in the timing of the lateral signal and found this not to be the case. We discovered that in order to adopt two different cell fates in two different executions, a VPC has to send the lateral signal before its neighbors in one execution, and after its neighbors in another execution. In the first case, the VPC will adopt a 1° fate and force its neighbors to adopt a 2° fate, while in the second case it is forced by one of its neighbors to adopt a 2° fate before it can adopt the 1° fate. Specifically, we found that in the four cases containing the *lin*-15(0) mutation, perturbation of the intricate timing dependency between the activation of the lateral signal and the inhibition of LIN-12 activity by the EGFR/RAS/MAPK pathway allows VPCs to adopt different fates in different executions of the model. Figure 6 distinguishes between stable and unstable fate patterns according to the ordering of events derived from the analysis of our model. In a stable pattern, the response to the inductive signal is temporally graded in a way that allows one VPC (e.g., VPC1 in Figure 6A) to send the lateral signal always before its neighbors reduce their level of LIN-12. In unstable patterns, on the other hand, the activation of the EGFR/RAS/MAPK pathway occurs more or less simultaneously in all VPCs, and small, stochastic timing differences result in variable patterns among genetically identical animals (Figure 6B). We note that this instability comes into effect only in AC-ablated animals or in VPCs that are too distant from the AC, suggesting that the AC organizes not only the spatial but also the temporal order of events.

**Figure 6:**
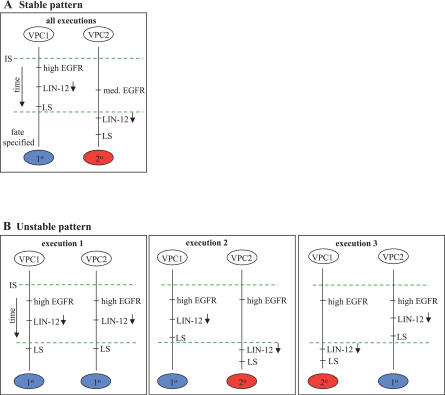
Order of Events in Stable and Unstable Fate Patterns Time flows from top to bottom. Two events that appear on the same vertical line are ordered according to the time flow. The dashed lines synchronize the different vertical lines. All events that appear above a synchronization line occur before all events that appear below the synchronization line. The time-order between two events that appear on parallel vertical lines without a synchronization line is unknown. (A) Proposed sequence of events leading to a stable pattern. The left time line starts with a high inductive signal (IS) and the right time line with a medium IS. (B) Three diagrams that represent possible sequences of events leading to different fate patterns in the absence of IS (the AC is absent). Execution 1 represents the case where two cells are strongly coupled and they both reduce their *lin*-12 level simultaneously, send LS, which is ignored, and assume primary fates. Execution 2 represents the case where the left cell sends the lateral signal slightly before its neighbor reduces the level of *lin*-12, thus resulting in a 1°-2° pattern. Execution 3 is the dual of execution 2 where the cell on the right inhibits the cell on the left.

### Experimental Validation of the Predictions: Loss of Sequential Induction in *lin*-15 Mutants

To test the predictions made by our model, we examined the expression of cell fate-specific transcriptional reporters in developing animals. Using a strain carrying both the *egl*-17::*cfp* and *lip*-1::*yfp* transgenes as reporters for the 1° and 2° cell fate, respectively, we could simultaneously observe the activation of the inductive and lateral signaling pathways in the VPCs of individual animals. We first performed a time-course analysis in a wild-type background and quantified the strength of the fluorescent signals of the 1° and 2° fate-specific markers during the critical phase from the mid L2 stage on (22 h after starvation-induced L1 arrest) until the end of the L2 stage just before the VPCs have adopted their fates and start dividing (28 h after starvation-induced L1 arrest). In all animals except for one case at the 25-h time point, an increase in the expression of the 1° fate marker *egl*-17::*cfp* was observed in P5.p, P6.p, and P7.p before a significant upregulation of the 2° fate marker *lip*-1::*yfp* occurred in P5.p and P7.p (Figure 7A, 7B, and 7D). Thus, the inductive signaling pathway is activated already in mid-L2 larvae (+22 h), while lateral signaling is effective only toward the end of the L2 stage (+28 h). These experimental data provide, for the first time to our knowledge, direct evidence for a sequential activation of the inductive and lateral signaling pathways during vulval induction, as predicted by our model in the case of stable fate patterns. It should be noted that mosaic analysis of *let*-23 *egfr* had already suggested a sequential model for vulval fate specification [24], though the relative timing of the inductive versus lateral signaling events has to date not been investigated.

**Figure 7:**
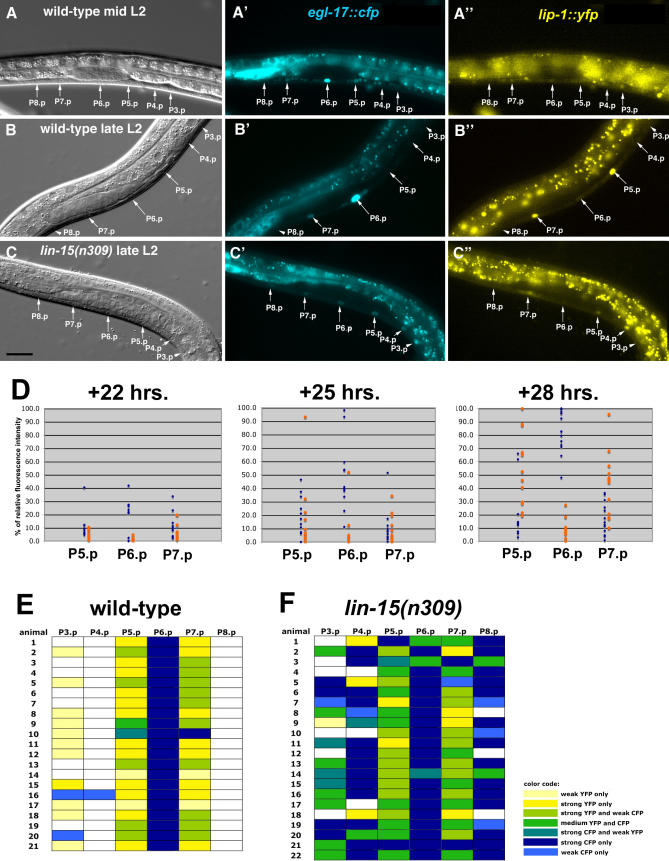
Experimental Validation of the Model's Predictions (A,B) As examples for the time-course analysis, a mid-L2 larva at +22 h (A–A″) and a late L2 larva at +28 h (B) are shown. For each animal, the Nomarski, CFP (‘), and YFP (‘') channels are shown. (C–C″) Example of a *lin*-15*(n*309) larva at the late L2 stage showing simultaneous expression of EGL-17::CFP and LIP-1::YFP in P7.p, P5.p, and P4.p. All images were taken with identical camera and microscope settings. Scale bar in C is 10 μm. (D) Quantification of the EGL-17::CFP (blue dots) and LIP-1::YFP (orange dots) signals in P5.p, P6.p, and P7.p of ten to 12 animals for each time point. The relative fluorescence intensities are shown as percent values of the maximal EGL-17::CFP and LIP-1::YFP signals, respectively, observed during the time-course analysis. (E,F) Semiquantitative representation of the EGL-17::CFP and LIP-1::YFP expression patterns observed in the VPCs of wild-type (E) and *lin*-15(*n*309) (F) late L2 larvae. Signal intensities were classified as indicated by the color legend on the right.

Next, we tested if in *lin*-15(*n*309) mutants that exhibit an unstable fate pattern in the distal VPCs the sequential activation of signaling pathways may be disrupted. Since larval development in *lin*-15(*n*309) animals is significantly delayed (unpublished data), it was not possible to perform the same time-course analysis as shown above for wild-type animals. We therefore staged *lin*-15(*n*309) animals carrying the *egl*-17::*cfp* and *lip*-1::*yfp* reporters based on the length of their posterior gonad arms and the shape of the VPCs to identify late L2 larvae corresponding approximately to the +28 h time point in wild-type larvae (see Materials and Methods). In 12 out of 22 late L2 *lin*-15(*n*309) larvae, the 1° and 2° fate markers were simultaneously expressed in at least one of the distal VPCs, P3.p, P4.p, or P8.p, which is consistent with the unstable fate pattern predicted for the distal VPCs in *lin*-15 mutants (Figure 7C and 7F). Moreover, in 21 out of 22 *lin*-15(*n*309) animals, P5.p and/or P7.p expressed both 1° and 2° fate markers (Figure 7F). In wild-type animals, on the other hand, co-expression of the 1° and 2° fate markers was never observed in the distal VPCs, but 12 out of 21 animals showed weak 1° fate marker expression in P5.p and/or P7.p in addition to the strong 2° marker expression (Figure 7E).

Thus, we could experimentally confirm two key predictions provided by our modeling work (Figure 6); the temporal gradient in the activation of the inductive and lateral signaling pathways in wild-type animals and the loss of sequential signaling in *lin-*15 mutants leading to an unstable fate pattern.

## Discussion

Formal executable models have become valuable tools to enhance our understanding of complex biological systems [1,3,25–30]. Here, we present an up-to-date comprehensive model of C. elegans vulval fate specification and experimental validation of two key predictions made by the model. Our model represents the current understanding of the regulatory signaling network and includes multiple modes of crosstalk between the EGFR/RAS/MAPK and NOTCH signaling pathways such as the LIN-12 / Notch-mediated lateral inhibition [9,10,22]. Since the model is dynamic and nondeterministic, it allows a very large number of different executions for a given starting condition. By using model checking, which permits us to investigate all possible executions of the model, we identify gaps in the conceptual understanding of the events leading to a stable pattern of vulval cell fates. The insights gained through model checking can then be used to refine an initial model until it fits all the experimental data. There could be several different ways to refine a model, and every conjecture made in the refinement process should then be validated experimentally. For example, our model suggests that the EGFR/RAS/MAPK pathway not only represses *lin-12/Notch* signaling [18] but also negatively regulates *lst* gene expression in 1° cells. In the 2° cells, on the other hand, lateral signaling overrides this postulated negative loop and *lst* activity prevents 1° cell fate specification. Although such a molecular mechanism has not yet been elucidated, our modeling study makes explicit the importance of this putative negative feedback loop. Interestingly, it was previously reported that some *lst* genes are not only positively regulated by lateral signaling but are also negatively regulated by inductive signaling [10]. In addition, the recently discovered homolog of the mammalian tumor suppressor *dep*-1 gene might also be part of this postulated negative feedback loop [31]. DEP-1 dephosphorylates the EGFR and thereby inhibits inductive signaling in the 2° cell lineage in parallel with the *lst* genes, while inductive signaling simultaneously downregulates DEP-1 and LIN-12/Notch expression in the 1° cell lineage, allowing full activation of the EGFR in these cells [18,31]. Thus, the reciprocal activation of EGFR/RAS/MAPK signaling and *lateral inhibitors* in 1° and 2° VPCs, respectively, might in part be mediated by a novel negative feedback loop downstream of the MAP kinase.

In mammals, the negative crosstalk between EGFR and Notch signaling may be important to control the balance between stem cell proliferation and differentiation [32], and alterations in the connections between these two signaling pathways may lead to cancer in humans [11]. Thus, future studies investigating the molecular details of negative feedback loops between EGFR signaling and the *lst* genes may help elucidate conserved mechanisms underlying EGFR function as an oncogene.

Our computational model allows flexibility in the order between different reactions, which resembles variations in the rate of biochemical reactions. This is akin to the robustness of simple biochemical networks that are resistant to variations in their biochemical parameters [33–35]. Despite this variability, we have found by model checking that in a wild-type situation all possible executions reach a stable state, independently of the order of reactions between the VPCs. This behavior of the model closely resembles the remarkable robustness of vulval development observed under various experimental conditions in the laboratory as well as in free living Nematodes. Furthermore, we observed that for most perturbations (i.e., mutations in the inductive or lateral signaling pathways), all the different executions lead to stable fate patterns. This suggests that the mechanism underlying VPC specification is relatively resistant to genetic variability and might therefore represent a process subject to high selective pressure.

A notable exception is the behavior of *lin*-15(*lf*) mutants, which—both experimentally and by modeling—exhibit an unstable fate pattern as long as the inductive signaling pathway is functional. We could trace down the cause of this instability to the fact that *lin*-15 mutations abrogate the temporal order in the activation of the inductive versus lateral signaling pathway among individual VPCs. Interestingly, recent experiments have demonstrated that *lin*-15(*lf*) mutations result in the ectopic expression of the inductive LIN-3 EGF signal in the hypodermal syncytium hyp7 [15]. Since all VPCs are in direct contact with hyp7, it seems reasonable to assume that in *lin*-15 mutants the EGFR is simultaneously activated in all VPCs, which likely disrupts the relative timing of inductive versus lateral signaling among adjacent VPCs. Our analysis of the behavior of *lin-15* mutants thus illustrates how computational modeling can provide a plausible mechanistic explanation for phenotypic instability observed in real life.

Through model checking an executable model representing the crosstalk between EGFR and LIN-12 / Notch signaling during C. elegans vulval development, we have gained new insights into the usage of these conserved signaling pathways that control many diverse processes in all animals. While many modeling efforts use simulations that allow us to investigate only a few possible executions, our work emphasizes the power of analyzing *all* possible executions using model checking. Previous attempts to use model checking in biological modeling have concentrated on adapting model checking to formalisms such as differential equations and probabilistic modeling [36–38]. Our work demonstrates how biological processes can be described and analyzed with the use of formal methods, which enhances our comprehension of complex biological systems. We suggest that combining model-checking analysis with high-level modeling, similar to the level of abstraction used by biologists in describing mechanistic models, can help in many areas of biology to obtain more accurate, formal, and executable models, eventually leading to better understanding of biological processes.

## Materials and Methods

### Reactive modules.

RM is a modeling language for reactive systems [20]. RM is designed to describe systems which are *discrete, deadlock-free,* and *nondeterministic*. The elementary particles in RM are *variables*. We describe the behavior of variables in *atoms* and combine atoms into *modules*. Modules can be combined to create more complicated modules (including combinations of several copies of the same module). Each variable ranges over a finite set of possible values. An atom describes the possible updates on variables. An atom can be *synchronous,* meaning that it updates the variables it controls in every step of the system, or *asynchronous*, meaning that it updates the variables it controls from time to time. An *update* of a variable may depend on the value of itself as well as the values of other variables. There can also be dependencies between the mutual update of several variables in the same step. RM enables nondeterminism by allowing multiple overlapping update options. The current RM model does not include probabilities.

### Mocha.

*Mocha* is a software tool for the design and analysis of RM [21]. Mocha can simulate a model by following step-by-step evolution of the variables in the model. Simulations show the sequence of values assumed by variables during the simulation. In simulation of nondeterministic models, the user is expected to choose the next step between different nondeterministic options. The simulation engine can highlight the variable values that lead to the assignment of a certain value. Mocha supports invariant model checking directly (to check that all reachable states satisfy some property that relates to the values of variables in the state), as well as model checking of safety properties using monitors (to check that all executions satisfy some property). We use both enumerative and symbolic (using Boolean Decision Diagrams, BDDs) model checking; the difference between the two has to do with their performance in practice. Counterexamples are presented as sequences of variable values.

### Modeling concurrency in biological systems.

Parallelism is an important property of biological systems. In computer science, this is referred to as *concurrency* (processes running in parallel and sharing common resources). We usually distinguish between two forms of concurrency: *synchronous* and *asynchronous*. In synchronous systems, all components move together. That is, there is some basic work unit that all components share. All components do one work unit in parallel simultaneously. Then they all move to the next unit. In asynchronous systems, every component moves separately. Usually, in asynchronous systems, we do not allow components to move together and we cannot guarantee the relative speed of different components. Biological systems, while highly concurrent, are neither completely synchronous nor completely asynchronous. Different molecules, or cells, do not progress in perfect lockstep, and neither does any molecule or cell rest for arbitrary amounts of time. For this reason, we have introduced a new notion of *bounded-asynchrony* into our computational model. In bounded-asynchrony, the scheduler, which chooses the next component to move, is not completely free in its choices. No component can be neglected more than a bounded number of times. This captures the phenomenon that the components of a biological system (say, molecules or cells, depending on the level of modeling granularity) progress neither in lockstep nor completely independently, but that they are loosely coupled and proceed approximately along the same timeline. We find the notion of bounded asynchrony a pragmatic way to model cell–cell interactions in an abstract discrete framework. Further studies are needed to identify the appropriate model for concurrency in different biological contexts.

### C. elegans microscopy and image analysis.

Standard methods were used for maintaining and manipulating C. elegans [39]. The *C. elegans Bristol* strain, variety N2, was used as the wild-type reference strain in all experiments. Mutations used: *lin*-15(*n*309) [22]; integrated transgene arrays used: *arIs*92[*egl*-17::*cfp,tax*-3::*gfp*] [10], *mfIs*42[*lip-1::yfp*] (gift of M. A. Félix).

Synchronized populations of L1 larvae were obtained by isolating embryos from gravid adults using sodium hypochlorite treatment and arresting the newly hatched larvae by food starvation. The arrested L1 larvae were then placed on standard NGM growth plates containing E. coli OP50 and collected for microscopic observation at the indicated time points. For observation under Nomarski optics, animals of the indicated stages were mounted on 4% agarose pads with M9 buffer containing 10 mM sodium azide. Fluorescent images were acquired on a Leica DMRA wide-field microscope equipped with a cooled CCD camera (Hamamatsu ORCA-ER, http://www.hamamatsu.com/) controlled by the Openlab 3.0 software package (Improvision, http://www.improvision.com/). For quantification of YFP and CFP intensity in the VPCs, all images were acquired with the same microscopy, camera, and software settings using YFP- and CFP-specific filter sets. The mean intensity of CFP and YFP expression in the nuclei of the VPCs was measured using the measurement tool in the Openlab 3.0 software package (Improvision), and each measurement was standardized to the background intensity in the same picture. For each time point, between ten and 12 animals were quantified. Late L2 *lin*-15(*n*309) animals were identified by selecting larvae in which the VPCs had adopted an oval shape and the distal tip of the posterior gonad arm had migrated past P7.p (see Figure 7B and 7C).

## Abbreviations

AC: gonadal anchor cell
EGFR: epidermal growth factor receptor
**lst**: lateral signal target
RM: reactive modules
synMuv: synthetic Multivulva
VPC: vulval precursor cell
